# Acute Splenic Sequestration Crisis in Hemoglobin SC Disease: Efficiency of Red Cell Exchange

**DOI:** 10.7759/cureus.12224

**Published:** 2020-12-22

**Authors:** Anjanaa Vijayanarayanan, Ayodeji J Omosule, Hannan Saad, Vrushali Dabak, Zaher K Otrock

**Affiliations:** 1 Pathology, Henry Ford Hospital, Detroit, USA; 2 Anesthesiology, Henry Ford Hospital, Detroit, USA; 3 Radiology, Henry Ford Hospital, Detroit, USA; 4 Hematology/Oncology, Internal Medicine, Henry Ford Hospital, Detroit, USA; 5 Pathology and Laboratory Medicine, Henry Ford Hospital, Detroit, USA

**Keywords:** red cell exchange, acute splenic sequestration, hemoglobin sc disease, adult, case

## Abstract

Acute splenic sequestration crisis (ASSC) is recognized as a serious complication of sickle cell disease in children. ASSC presents with progressive splenic enlargement, transfusion-dependent anemia, and, eventually, circulatory compromise. ASSC is rare in adult patients, thus making its management and outcome in adults not well-defined. The purpose of this article is to describe our experience in managing ASSC in an adult female with hemoglobin (Hb) SC disease. The patient underwent an automated red blood cell (RBC) exchange, thus avoiding a planned splenectomy. To the best of our knowledge, our case is the third report in the literature on the use of RBC exchange in adults with HbSC disease and ASSC. RBC exchange should be considered in adults with HbSC disease with ASSC not responding to simple transfusion; a treatment that could alleviate patients' symptoms and avoid splenectomy complications, especially in young patients.

## Introduction

One of the earliest life-threatening complications of sickle cell disease (SCD) is acute splenic sequestration crisis (ASSC). ASSC is defined as acute splenic enlargement with a drop in hemoglobin (Hb) level by at least 2 g/dL [1]. ASSC is primarily seen in young children with sickle cell anemia and less commonly with hemoglobin SC (HbSC) disease. Children with homozygous sickle cell disease (HbSS) are often affected until the age of six while individuals with other heterozygous forms of sickle cell disease (HbSβThal, HbSC, HbSD) are at risk even into adulthood [2]. While the prevalence of ASSC in children with HbSS is fairly high ranging from 7% to 30% according to studies [1,3-4]. ASSC is quite rare in adult patients with HbSC disease and manifests later in life, possibly as late as the eighth decade [5].

ASSC occurs due to the rapid sequestration of red blood cells in the splenic red pulp, sudden enlargement of the spleen (within hours), a precipitous decline in Hb and platelets, and an increase in reticulocytes. ASSC in adults most often presents with symptoms such as left upper quadrant abdominal pain, left flank pain, left-sided pleuritic chest pain, fever, chills, and profound weakness [5]. The management of splenic dysfunction in HbSC patients has been extended from studies done on HbSS without an evidence‐based rationale. Most often, therapy consists of red blood cell (RBC) transfusion, fluid resuscitation, and splenectomy [6].

Simple RBC transfusions are recommended in SCD when symptoms are primarily due to anemia with Hb level < 9 g/dL, whereas exchange transfusions (RCE; red cell exchange) are indicated to avoid or treat complications of SCD due to HbS with or without HbC [7]. The majority of automated RCEs are performed for the prevention or treatment of complications. Splenectomy is often preferred especially in recurrent ASSC and if the patient with a history of multiple transfusions has developed several alloantibodies [8]. Here, we discuss the favorable outcome of ASSC in an adult female with HbSC disease managed with RBC exchange, thus avoiding a scheduled splenectomy.

This case was presented during the American Society for Apheresis (ASFA) 39th Annual Meeting, Chicago, Illinois, April 25-28, 2018 [9].

## Case presentation

**Figure 1:**
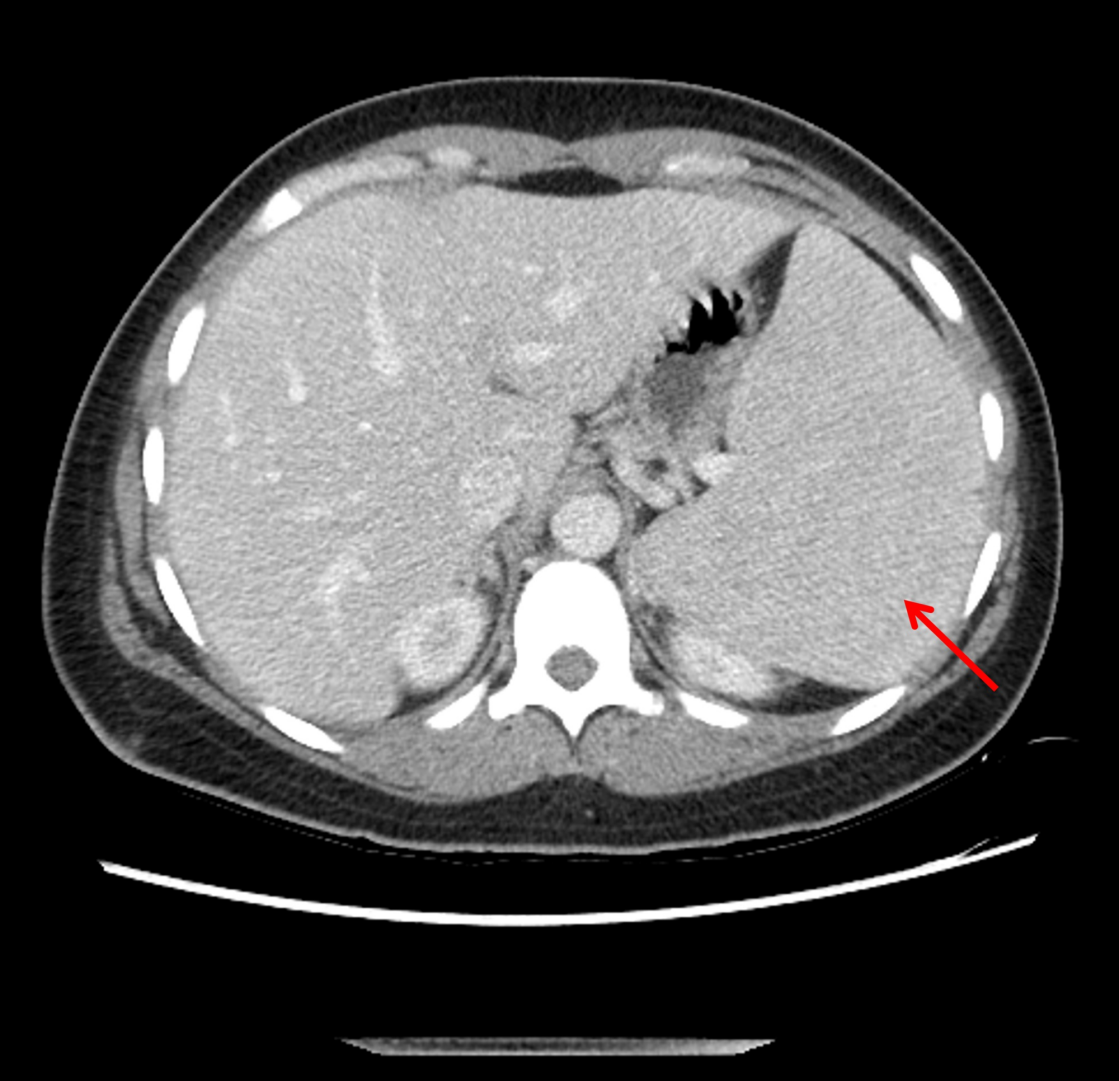
Computed tomography scan of the abdomen demonstrating splenomegaly (red arrow)

A 39-year-old African American lady with a history of HbSC disease, diagnosed at the age of 10 years, presented to the surgical outpatient clinic with progressive abdominal pain and swelling of one-month duration. Her symptoms were associated with early satiety for which she had lost some weight. Her examination revealed a markedly enlarged spleen measuring 20 cm in its longest dimension on computed tomography (CT) scan, raising the suspicion for ASSC (Figure 1).

She denied chest pain or dyspnea. As a result, she has been transfused with multiple packed red blood cell (RBC) units over the past month without improvement of her symptoms. She has presented with abdominal pain and splenomegaly eight months prior to the current admission; her symptoms were mild and she was managed with hydration and pain medications.

Splenectomy was recommended and planned due to the persistence of the patient’s symptoms. The transfusion medicine service was consulted to perform one session of automated RBC exchange before splenectomy. Upon evaluation, the patient was noted to be in severe pain with a numerical rating scale of 8/10. On examination, she had left upper quadrant tenderness and swelling with appreciable splenomegaly. The patient’s laboratory values on admission were white blood cell (WBC) 3.8×109/L, Hb 8.7 g/dL, hematocrit 25.9%, mean corpuscular volume (MCV) 81.2 fL, and platelets 105×109/L. It was noted that her Hb and platelet counts were 10.2 g/dL and 154×109/L, respectively, 10 days before admission. Hb electrophoresis analysis revealed a combined Hb S and C of 91%.

A central venous catheter was placed in the right internal jugular vein for vascular access. The patient’s antibody screen was positive for anti-C, -E, and -Fy(a). After obtaining informed consent, RBC exchange was performed with the COBE Spectra apheresis system (COBE Spectra, Terumo BCT, Inc., Lakewood, Colorado) processing two blood volumes and replacing with fresh (<10 days old), leukoreduced, sickle-cell negative, C, E, K and Fy(a) antigen-negative packed RBCs. Anticoagulant citrate dextrose solution, solution A (ACD-A) was used, 3 g of IV calcium gluconate was infused throughout the procedure, and 25 mg IV Benadryl was given to treat allergic reactions We aimed for end hematocrit of 30% and end Hb S+C of 20%. We considered the possibility that the ASSC would respond to RCE without the need for splenectomy. The case was discussed with the surgical team, and we agreed to hold splenectomy to evaluate the patient’s response to RBC exchange. The patient tolerated RCE well; her post-procedure period was complicated by hypotension and tachycardia, which was managed with adequate hydration.

**Figure 2:**
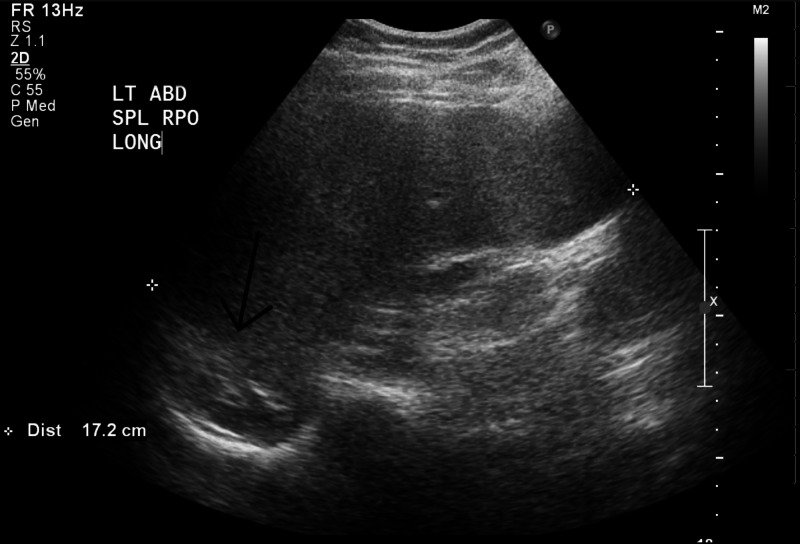
Long-axis gray-scale ultrasound image of the left upper quadrant in the right posterior oblique position demonstrates splenomegaly of up to 17 cm in the craniocaudal dimension Additionally, there is evidence of mass effect on the adjacent left kidney (black arrow).

Repeat evaluation after RCE revealed a Hb of 9.1 g/dl and Hb electrophoresis showed Hb S+C of 29.8%. The patient reported feeling better after the RBC exchange, and she could eat well without discomfort. Her pain scale was 2/10. Her spleen was barely palpable below the left costal margin, and there was a reduction in splenic size on abdominal ultrasound imaging to 17 cm four days after RCE (Figure 2).

On the last follow-up one year after the RBC exchange, the patient was doing well. She had an episode of pain crisis seven months after her last ASSC and was managed with manual exchange transfusion. Repeat imaging seven months after the RBC exchange re-demonstrated enlarged spleen measuring 16 cm in its longest dimension. The patient never reported abdominal pain and did not have any recurrence of ASSC.

## Discussion

In the United Kingdom and United States, HbSC accounts for 30% of SCD [10]. The red blood cells in HbSC disease contain equal amounts of both HbS and HbC. Although individuals with either HbS or HbC traits are rarely symptomatic, the combination of these two hemoglobins (i.e. HbSC disease) results in a moderately severe disease phenotype that manifests with frequent complications [11]. Koduri and colleagues reviewed 106 adult patients with HbSC disease and identified the following clinical features: splenomegaly (36%), proliferative sickle retinopathy (34%), avascular necrosis of the hip (23%), and hematuria (20%) [12]. ASSC was diagnosed in 10 (9.4%) patients and was the presentation of SCD in two of these patients. Their study does not mention how these patients were managed.

Splenic complications constitute one of the major morbidities in HbSC patients. Functional asplenia has been reported in up to 45% of HbSC patients [13]. ASSC is commonly observed in children with HbSS disease. In contrast, adult HbSS patients would have their spleen scarred and atrophied due to the recurrent sickling episodes and subsequent splenic infarction during childhood. Thus, they rarely experience ASSC. HbSC patients often maintain their splenic function and are thus at risk of ASSC [14]. ASSC can be one of the manifestations of splenomegaly in these patients; it is precipitated by the outflow of sickled and damaged RBCs causing obstruction of splenic sinusoids. ASSC can occur in 10% of adult HbSC [12]. HbSC patients remain at risk of ASSC even into their 8th decade. Although relatively uncommon, ASSC can be a serious and occasionally fatal complication [5].

Although fever has been considered characteristic of ASSC in previous reports, recent data suggest that fever is not very common and may not play a significant role in diagnosis. Naymagon et al. reported on 10 adult HbSC patients with ASSC among whom only one patient presented initially with fever and another four patients developed fever during hospital admission [8]. In addition, most of the patients’ hospital course was characterized by hemodynamic instability manifesting with hypotension, tachycardia, or orthostasis [8].

Although there are no clinical trials on ASSC management, most reported studies have agreed that RBC transfusion is the primary treatment for ASSC. RBC transfusion must be guided by clinical and laboratory parameters, as entrapped RBCs in the spleen may be mobilized, thus increasing the patient’s hemoglobin more than expected. The usual management of sickle cell crisis with supportive measures and hydration should also be instituted. Splenomegaly often improves after RBC transfusion. Resistant or recurrent ASSC might be managed with splenectomy [5,8,15]. Total splenectomy will prevent splenic sequestration crises. The risks of splenectomy should be discussed with the patient; these include procedure-related complications, increased susceptibility to encapsulated bacterial infections, and fulminant sepsis.

The role of RBC exchange in adults with HbSC disease and ASSC has not been elucidated. Our review of the literature yielded only two cases treated with RBC exchange. Squiers et al. reported on a 70-year-old female with HbSC disease who presented with ASSC complicated with acute chest syndrome [16]. The patient was managed in the medical intensive care unit and was treated with simple RBC transfusions initially then an RBC exchange, following which she had a relatively uncomplicated hospital course. In another case series reviewing adult ASSC institutional experience, 10 patients with HbSC were presented [8]. Seven patients were treated with simple transfusions, one with simple and exchange transfusions, and two did not require transfusion. Two of the patients who received simple transfusions underwent splenectomy. The only patient who received RBC exchange was a 26-year-old female who presented with left arm pain without fever. From what has been described in the report, she developed a fever during admission, did not have hemodynamic instability, received two units of packed RBCs without response, and then underwent RBC exchange. She previously had two similar episodes of ASSC [8].

## Conclusions

Our case serves to illustrate the efficacy and favorable outcome of automated RBC exchange in an adult HbSC patient with ASSC, thus avoiding a scheduled splenectomy. Our patient was a young female who presented with ASSC and did not respond to simple blood transfusion. It is possible that our patient had chronic splenic sequestration that was complicated by ASSC. She had a history of splenomegaly, and her spleen size has persisted following RCE despite the improvement in her acute symptoms. Her clinical presentation with severe abdominal pain and sudden drop in Hb was consistent with ASSC. Hb electrophoresis was very helpful in managing our patient, as it revealed high levels of Hb S and C despite the patient’s previous transfusions. This was an indication of an aggressive crisis and prompted us to consider automated red cell exchange as a more efficient treatment.

To the best of our knowledge, our case is the third report in the literature on the use of RBC exchange in adults with HbSC disease and ASSC. Automated RBC exchange seemed an effective treatment for ASSC and provided good clinical response. More importantly, we spared the patient’s spleen and all potential complications and risks of splenectomy.
